# Multi-omics analysis explores the effect of chronic exercise on liver metabolic reprogramming in mice

**DOI:** 10.3389/fcell.2023.1199902

**Published:** 2023-06-20

**Authors:** Zhaoxu Lu, Ping Qian, Jiahui Chang, Xuejia He, Haifeng Zhang, Jian Wu, Ting Zhang, Jianxin Wu

**Affiliations:** ^1^ Children’s Hospital Capital Institute of Pediatrics, Chinese Academy of Medical Sciences and Peking Union Medical College, Beijing, China; ^2^ Beijing Municipal Key Laboratory of Child Development and Nutriomics, Capital Institute of Pediatrics, Beijing, China; ^3^ Graduate School of Peking Union Medical College, Beijing, China; ^4^ Beijing Municipal Key Laboratory of Child Development and Nutriomics, Capital Institute of Pediatrics-Peking University Teaching Hospital, Beijing, China; ^5^ Beijing Municipal Key Laboratory of Child Development and Nutriomics, Experimental Center, Capital Institute of Pediatrics, Beijing, China; ^6^ School of Kinesiology and Health, Capital University of Physical Education and Sports, Beijing, China; ^7^ Beijing Tongren Hospital, Capital Medical University, Beijing, China

**Keywords:** chronic exercise, transcriptome, proteome, acetyl-proteome, metabolome, liver

## Abstract

**Background:** The effect of exercise on human metabolism is obvious. However, the effect of chronic exercise on liver metabolism in mice is less well described.

**Methods:** The healthy adult mice running for 6 weeks as exercise model and sedentary mice as control were used to perform transcriptomic, proteomic, acetyl-proteomics, and metabolomics analysis. In addition, correlation analysis between transcriptome and proteome, and proteome and metabolome was conducted as well.

**Results:** In total, 88 mRNAs and 25 proteins were differentially regulated by chronic exercise. In particular, two proteins (Cyp4a10 and Cyp4a14) showed consistent trends (upregulated) at transcription and protein levels. KEGG enrichment analysis indicated that Cyp4a10 and Cyp4a14 are mainly involved in fatty acid degradation, retinol metabolism, arachidonic acid metabolism and PPAR signaling pathway. For acetyl-proteomics analysis, 185 differentially acetylated proteins and 207 differentially acetylated sites were identified. Then, 693 metabolites in positive mode and 537 metabolites in negative mode were identified, which were involved in metabolic pathways such as fatty acid metabolism, citrate cycle and glycolysis/gluconeogenesis.

**Conclusion:** Based on the results of transcriptomic, proteomics, acetyl-proteomics and metabolomics analysis, chronic moderate intensity exercise has certain effects on liver metabolism and protein synthesis in mice. Chronic moderate intensity exercise may participate in liver energy metabolism by influencing the expression of Cyp4a14, Cyp4a10, arachidonic acid and acetyl coenzyme A and regulating fatty acid degradation, arachidonic acid metabolism, fatty acyl metabolism and subsequent acetylation.

## 1 Introduction

Physical activity or exercise training is important not only for mental health but also for physical health and play major roles in the overall health status of humans. It has been reported that exercise can serve as a treatment for various diseases, including psychiatric diseases, cardiovascular diseases, neurological diseases, musculo-skeletal disorders, metabolic diseases, and cancer (Pedersen and Saltin, 2015). A review concluded that the cholesterol levels and blood lipids can be influenced by regular activity (Mann et al., 2014). Extensive studies have highlighted that increased physical exercise can significantly improve the glucose control in patients with type 2 diabetes and type 1 diabetes (Balducci et al., 2014; Riddell et al., 2017; Lu and Zhao, 2020; Kanaley et al., 2022). Fats and carbohydrates are the most important energy sources for exercise and physical activity. A review in 2015 delineated the effects of exercise on carbohydrate metabolism in skeletal muscle and systemic glucose homeostasis (Mul et al., 2015). Aucouturier et al. (2008) summarized the factors that affect carbohydrate and lipid metabolism during exercise in children. Taken together, the effect of exercise on human metabolism is obvious.

The liver is the central organ that controls systemic metabolic homeostasis, which regulates a series of metabolic functions such as the metabolism of amino acids, fatty acids and carbohydrates through integrating the complex signals from the portal (e.g., nutrient-rich blood from the spleen or intestines) and systemic (i.e., arterial blood) circulation (Murphy et al., 2020). Under the control of a complex transcriptional network, the liver not only provides the necessary metabolic substrate, i.e., glucose derived from liver glycogen, for skeletal muscle contraction, but also produces triglyceride-rich very low-density lipoproteins to fulfill the increased demand for energy of the whole body and maintain systemic metabolic balance (Jang et al., 2012; Murphy et al., 2020). Although exercise has potential benefits effects on systemic metabolism, its biological mechanisms is not sufficiently known.

Advances in omics technologies have made it possible to carry out large-scale molecular analysis for biological systems. Single omics investigations make the dependencies between biological features and the relationships between different molecular layers still elusive (Agamah et al., 2022). Information at multiple omics levels can provide more evidence for biological mechanisms at different molecular layers and further understand the complex mechanisms of biological processes and complex phenotypes. Proteomics is a study of proteomes to identify significantly altered proteins that represent the contents of cells, tissues, organisms, or biofluids (Qian X. et al., 2022). Metabolomics is a subject to analyze qualitatively and quantitatively endogenous metabolites in cells, biofluids and/or tissues (Song et al., 2019).

Herein, we conducted transcriptomics, proteomic, acetyl-proteomics and metabolomic profilings to explore the effect of chronic exercise on liver metabolism in mice. The crucial proteins, metabolites and important pathways involved in exercise affecting liver tissue metabolism in mice were also determined.

## 2 Materials and methods

### 2.1 Experimental animals

Adult C57BL/6J male mice (20–22 g, 8 weeks), purchased from Charles River Laboratory, were used in this study. After 1-week adaptation, mice were randomly divided into two groups: the chronic exercise (Exe) and the sedentary control (Con). The mice were housed in a 12-h light/dark cycle (light on at 6:00 a.m.) in a temperature- and humidity-controlled condition (22°C ± 2°C) with free access to food and water. All animal procedures were conducted during rodents’ nocturnal phase (6:00 p.m.–12:00 p.m.). The animal experiments were complied with the institutional ethical guidelines and approved by the Ethics Committee on Animal Care and Use of the Capital Institute of Pediatrics (DWLL2021015).

### 2.2 Chronic exercise protocol

The moderate-intensity treadmill exercise was performed as previously described (Qian P. et al., 2022). The training stage lasted for 6 weeks, five consecutive days per week and 60 min per day. The assigned speed was 12 m/min for the first 2 weeks and added 1 m/min biweekly. The running distance for each mouse covered 22 km approximately in the training stage. The Con mice remained in the same room without access to the treadmills throughout the experiment.

### 2.3 Tissue collection

Mice from each group were euthanized and sacrificed by cervical dislocation within 24 h after the last exercise. The mouse chest was opened and the heart was perfused with saline until the liver turned white. Avoiding the major blood vessels and bile ducts at the lobe stem, liver tissues were then excised and frozen in liquid nitrogen and then stored at −80°C until assays. Principal component analysis (PCA) was performed to exclude the outliers. Transcriptomics and metabolomics analysis was performed on liver tissue samples from 12 mice (n = 6, both group). For proteomics and acetyl-proteomics analysis, liver tissue samples from 8 mice were used (n = 4, both groups).

### 2.4 Transcriptomics analysis

Total RNAs were isolated from liver tissue samples with TRIzol reagent. Based on the DNBseq platform (PE150 strategy), sequencing was performed. The clean reads were obtained from RNA-sequencing results by removing the sequence with low quality including adapter sequences, sequences with quality score <20, sequences with N base rate of raw reads >10% and sequence less than 25bp. HISAT was used to align clean reads with the reference genome GCF_000001635.27_GRCm39. Differential expression analysis was performed using the DESeq. Differentially expressed genes (DEGs) were obtained with |log_2_FC| > 1 and Q-value ≤ 0.05. David was used to perform GO and KEGG enrichment analysis for DEGs with *p*. adjust < 0.05.

### 2.5 Liquid chromatography-tandem mass spectrometry analysis

Liquid chromatography-tandem mass spectrometry was performed at PTM Bio lab (Hangzhou, China). Protein extraction, trypsin digestion, and acetylated peptide enrichment was performed as described by Qian P. et al. (2022). Liquid chromatography-tandem mass spectrometry was performed as previously described (Qian P. et al., 2022) with a minor modification of the following parameters: Peptides for proteome were separated with a gradient from 6% to 24% solvent B (0.1% formic acid in acetonitrile) over 70 min, 24%–35% in 14 min and climbing to 80% in 3 min then holding at 80% for the last 3 min. Peptides for acetylome were separated with a gradient from 7% to 24% solvent B over 40 min, 24%–32% in 12 min and climbing to 80% in 4 min then holding at 80% for the last 4 min. Database searching was performed as previously described with a minor modification of the following parameters: The mass tolerance for precursor ions was set as 20 ppm in first search and 20 ppm in mainsearch, and the mass tolerance for fragment ions was set as 20 ppm.

### 2.6 Proteomics and acetyl-proteomics analysis

Differentially expressed proteins (DEPs), differentially acetylated proteins (DAPs) and differentially acetylated sites (DASs) were determined with *p*-value < 0.05, and fold change (FC) cutoff as 1.5. The subcellular structure of the protein were annotated using WoLF PSORT software. GO and KEGG enrichment analysis was performed with *p*-value < 0.05.

### 2.7 Metabolomics analysis

The liver tissue sample stored at −80°C refrigerator was thawed on ice. The thawed sample was homogenized for 20 s, and then centrifuged at 3,000 rpm for 30 s (4°C). A 400 μL solution (Methanol: Water = 7:3, V/V) containing internal standard was added in to 20 mg grinded sample, and shaked at 1,500 rpm for 5 min. After placing on ice for 15 min, the sample was centrifuged at 12,000 rpm for 10 min (4°C). A 300 μL of supernatant was collected and placed in −20°C for 30 min. After centrifuged at 12,000 rpm for 3 min (4°C), a 200 μL aliquots of supernatant were transferred for LC-MS analysis. The metabolic profile was analyzed in both ESI-positive and ESI-negative ion modes. The Waters ACQUITY UPLC HSS T3 C18 (1.8 µm, 2.1 mm × 100 mm) was employed in both positive and negative modes. The LC-MS raw data were converted to an mzXML format by the ProteoWizard software. Data pretreatment was performed using package XCMS in R 3.5.1. The potential metabolite was appraised by searching the laboratory’s self-built database, integrated public database, AI database and metDNA. Differential metabolites (DEMs) were determined by variable importance value (VIP) > 1 with *p*-value < 0.05 based on a Student’s *t*-test and plotted by the ComplexHeatmap package in R. KEGG enrichment analysis was performed using KEGG database with *p*-value < 0.05.

## 3 Results

### 3.1 Chronic exercise altered the transcriptome of liver tissue in mice

**FIGURE 1:**
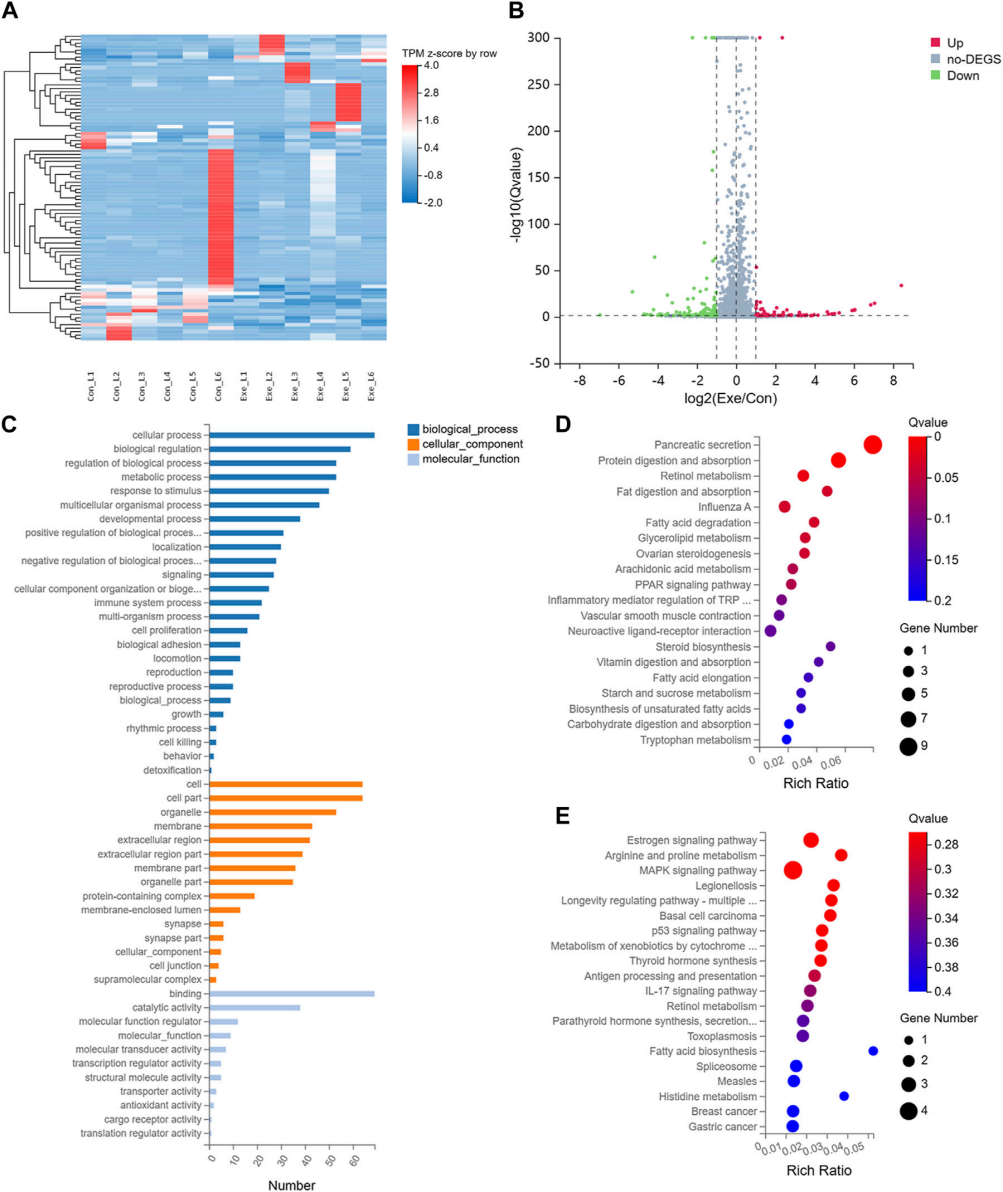
Transcriptomics analysis **(A)** Heatmap of the DEGs. **(B)** Volcano plot of the DEGs. **(C)** GO analysis for DEGs. **(D)** KEGG pathway annotation for upregulated DEGs. **(E)** KEGG pathway annotation for downregulated DEGs.

To investigate the effects of chronic exercise on the transcriptome of liver tissue in mice, transcriptomics analysis was performed. Compared with Con group, a total of 88 mRNAs, including 28 up- and 60 downregulated, were recognized as DEGs with |log_2_FC| > 1 and Q-value ≤ 0.05 in Exe group (Figures 1A, B). Functional enrichment analysis indicated that these 88 DEGs were involved in multiple metabolic pathways, including metabolism of cofactors and vitamins, amino acid metabolism, lipid metabolism and carbohydrate metabolism (Figures 1C–E). Specifically, these metabolic pathways include retinol metabolism (Cyp1a1, Cyp4a10, and Cyp4a14), fatty acid metabolism (fatty acid degradation (Cyp4a10 and Cyp4a14) and fatty acid biosynthesis), vitamin digestion and absorption, fat digestion and absorption, carbohydrate digestion and absorption, glycerolipid metabolism, and arachidonic acid metabolism (Cyp4a10 and Cyp4a14).

### 3.2 Proteomics changes

**FIGURE 2:**
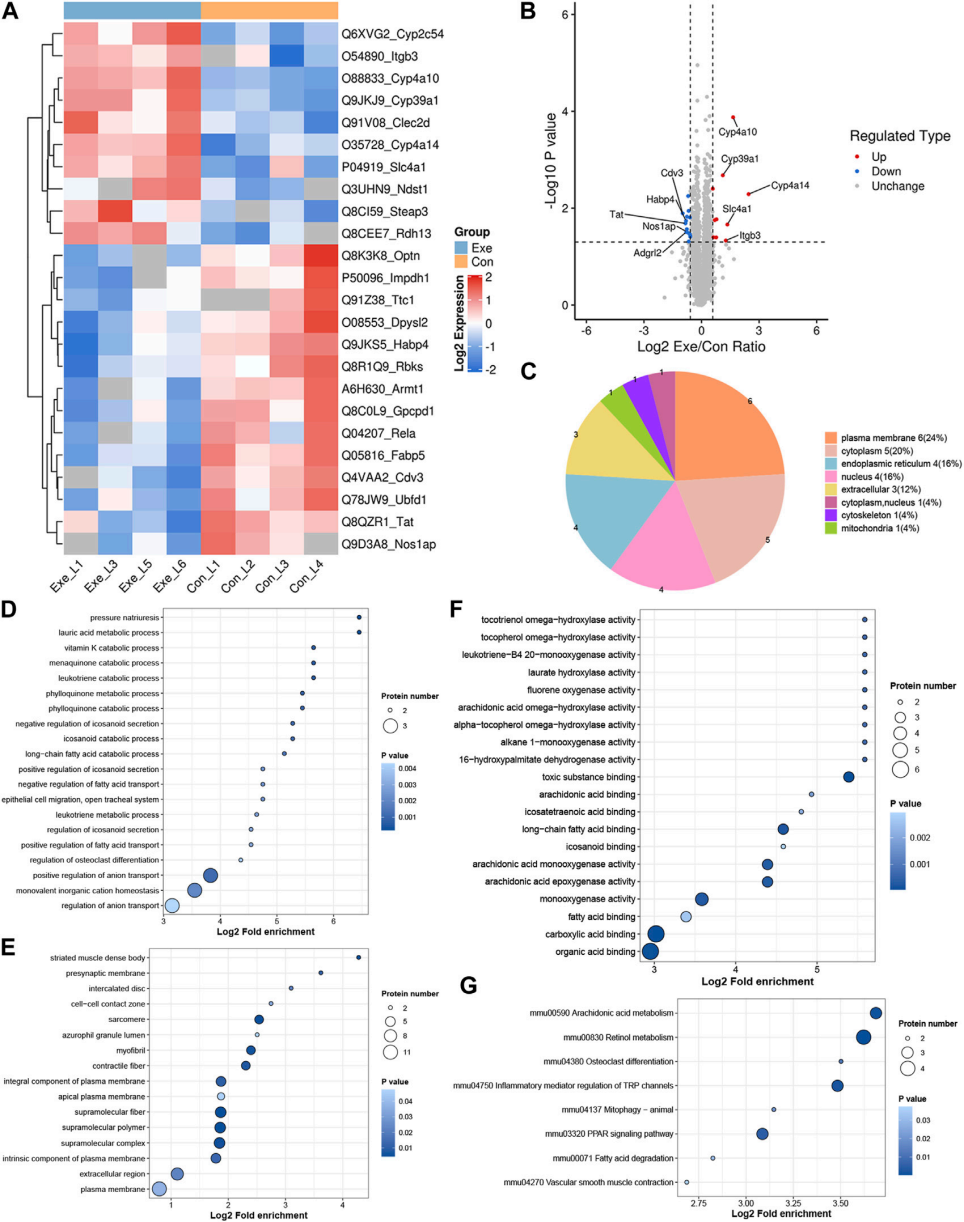
Proteomics analysis **(A)** Heatmap of the DEPs. **(B)** Volcano plot of the DEPs. **(C)**. Subcellular distribution for DEPs. **(D–F)** GO analysis for DEPs, **(D)** biological process, **(E)** cellular component, **(F)** molecular function. **(G)** KEGG pathway annotation for DEPs.

Due to post-transcriptional regulation as well as post-translational regulation, mRNA expression levels are not representative of protein levels. As the main executors of cell functions, proteins are the main effector molecules that ultimately participate in cell biological processes. Herein, proteomics analysis was further performed. Compared with Con group, a total of 25 DEPs, including 10 up- and 15 downregulated DEPs, were identified in Exe group (Figures 2A, B). These 25 proteins were predicted to distribute in virtually every subcellular compartment (Figure 2C). Similarly, functional enrichment analysis was performed for these 25 proteins (Figures 2D–G). Cyp4a14, Cyp4a10, Cyp2c54, and Rdh13 were involved in retinol metabolism pathway, Cyp4a14, Cyp4a10, and Cyp2c54 were involved in arachidonic acid metabolism and inflammatory mediator regulation of TRP channels pathway, and Cyp4a14 and Cyp4a10 were involved in fatty acid degradation, vascular smooth muscle contraction and PPAR signaling pathway. In addition, Rela and Optn were involved in mitophagy pathway. As an autophagy adaptor, Optn plays crucial roles in selective ubiquitination of damaged mitochondria (Yamano et al., 2020).

### 3.3 Correlation analysis between transcriptome and proteome

**FIGURE 3:**
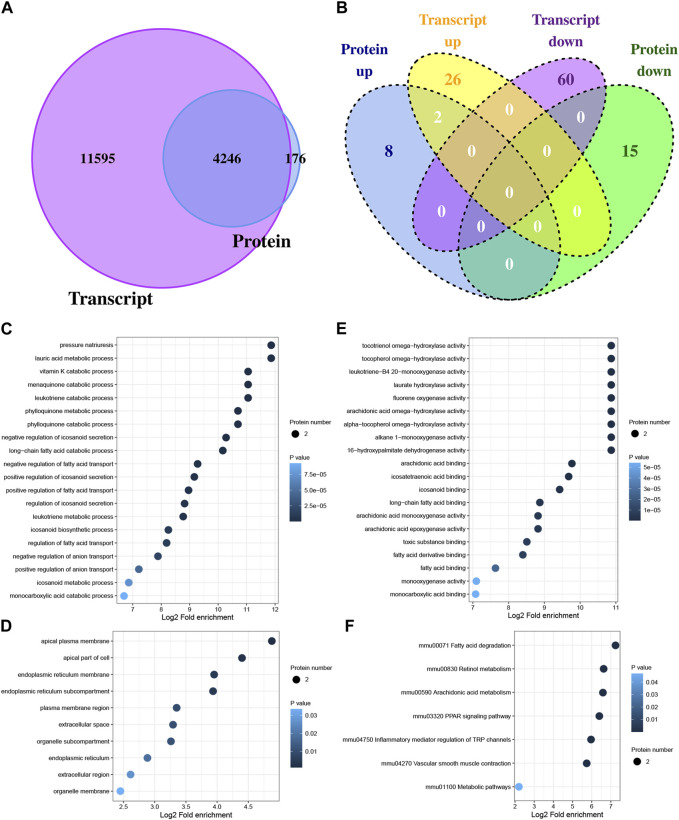
Correlation analysis between DEGs and DEPs **(A)** Venn diagram displaying the genes and protein quantified. **(B)** Venn diagram displaying the DEGs and DEPs. **(C–E)** GO analysis for proteins with concordant directions at both the mRNA and protein level, **(C)** biological process, **(D)** cellular component, **(E)** molecular function. **(F)** KEGG pathway annotation for proteins with concordant directions at both the mRNA and protein level.

The potential regulatory relationships between proteins and transcripts can be quickly understood by analyzing the correlation between transcriptome and proteome. In this study, 15,841 genes and protein quantified, and 4,246 genes were quantified at both the transcription level and the protein level (Figure 3A). In total, 88 DEGs and 25 DEPs were obtained between Con and Exe group. Furthermore, to determine the complementarity and integration of mRNAs and proteins, the concordance in the directions of change between mRNA and protein level was evaluated. As presented in Figure 3B, only two proteins (Cyp4a10 and Cyp4a14) exhibited the same expression trends (upregulated) at both the transcription level and the protein level, which revealed the widespread existence of active post-transcriptional and post-translational regulation. Functional enrichment analysis was performed for Cyp4a10 and Cyp4a14 (Figures 3C–F). The biological processes analyzed by GO for Cyp4a10 and Cyp4a14 were mainly involved in various metabolic pathways, such as, lauric acid metabolic process, menaquinone catabolic process, leukotriene catabolic process and vitamin K catabolic process (Figure 3C). KEGG enrichment analysisindicated that Cyp4a10 and Cyp4a14 are mainly involved in fatty acid degradation, retinol metabolism, arachidonic acid metabolism and PPAR signaling pathway (Figure 3F).

### 3.4 Identification of acetylation landscape in the mouse liver

**FIGURE 4:**
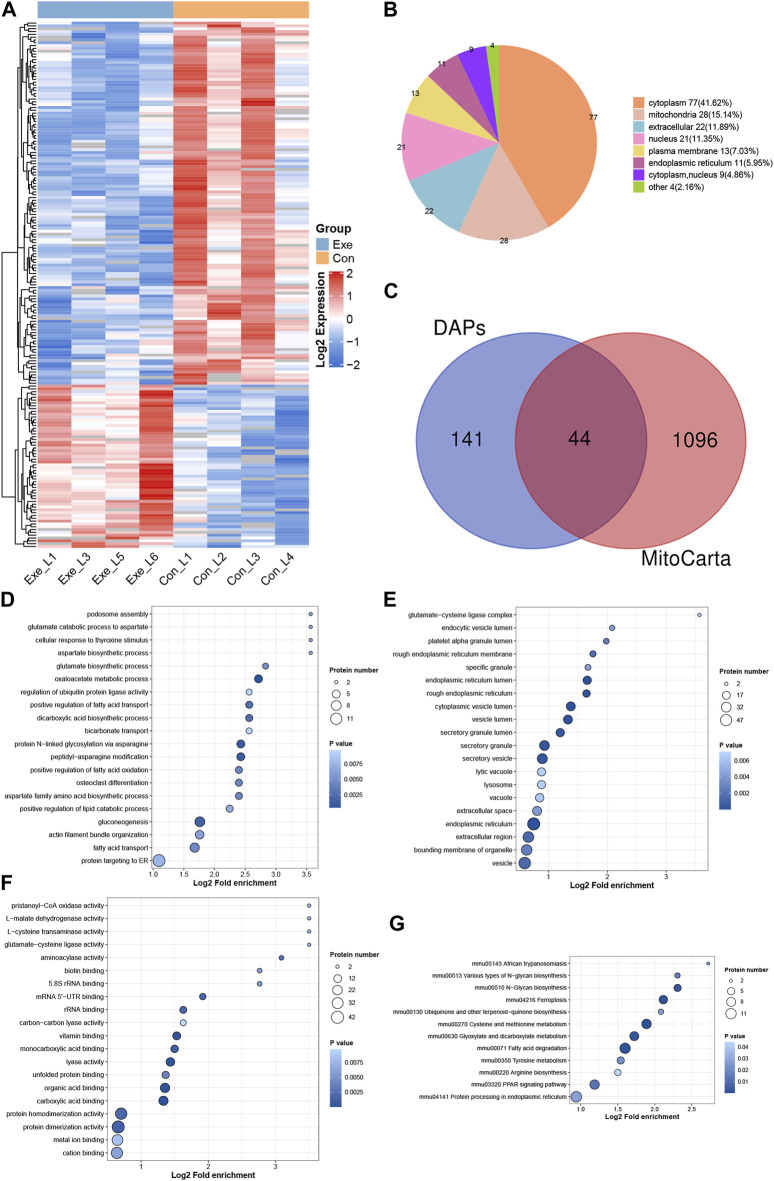
Acetyl-proteomics analysis **(A)** Heatmap of the DASs. **(B)** Subcellular distribution for DAPs. **(C)**. Venn diagram showing the overlap between the DAPs and proteins identified in MitoCarta. **(D–F)** GO analysis for DAPs, **(D)** biological process, **(E)** cellular component, **(F)** molecular function. **(G)** KEGG pathway annotation for DAPs.

Both transcriptomic and proteomic results demonstrated the effect of chronic exercise on liver metabolism in mice, prompting an investigation of acetylation. In total, 185 DAPs and 207 DASs (64 up- and 143 downregulated) were detected in acetyl-proteomics analysis (Figure 4A). These 185 proteins were predicted to distribute in virtually every subcellular compartment, with cytoplasm and mitochondria the most dominant localizations (Figure 4B). Among them, 44 proteins were mitochondrial proteome based on MitoCarta (Figure 4C). Functional enrichment analysis was performed for these 185 proteins (Figures 4D–G). KEGG analysis indicated that upregulated DAPs were significant enriched in fatty acid biosynthesis (Fasn, Acsl1 and Acsl5) and PPAR signaling pathway (Apoa2, Acsl1, Cpt1a, Acsl5 and Acox2), while downregulated DAPs were significant enriched in fatty acid degradation (Cyp4a12a, Cyp4a10, Adh5, Acadvl, Acat2, Adh4, Acox3, Ehhadh, Acsl1 and Acsl5), Citrate cycle (TCA cycle) (Mdh2, Mdh1, Aco1, Pc, and Aco2), retinol metabolism (Cyp4a12a, Cyp4a10, Adh4, Adh5, Rdh16, Ugt2a3), PPAR signaling pathway (Cyp4a12a, Cyp4a10, Ehhadh, Acox3, Acsl1 and Acsl5). Among them, Acsl1, Acadvl, Mdh2, Ehhadh and Aco2 involved in the above pathways were mitochondrial proteins.

### 3.5 Effect of chronic exercise on liver metabolites in mice

Previous study had indicated that some substances were easily detected in positive mode, whereas others were easily in negative mode (Ding et al., 2021). To expand the possibility of identification and determination of the metabolites in the samples, a combination of positive mode and negative mode was used in this study. Compared with Con group, 693 DEMs in positive mode and 537 DEMs in negative mode were identified. There was only one DEM, coenzyme A, identified in both two modes. The results of pathway analysis displayed that only one pathway, namely, fatty acid degradation, was enriched in both positive and negative modes.

**FIGURE 5:**
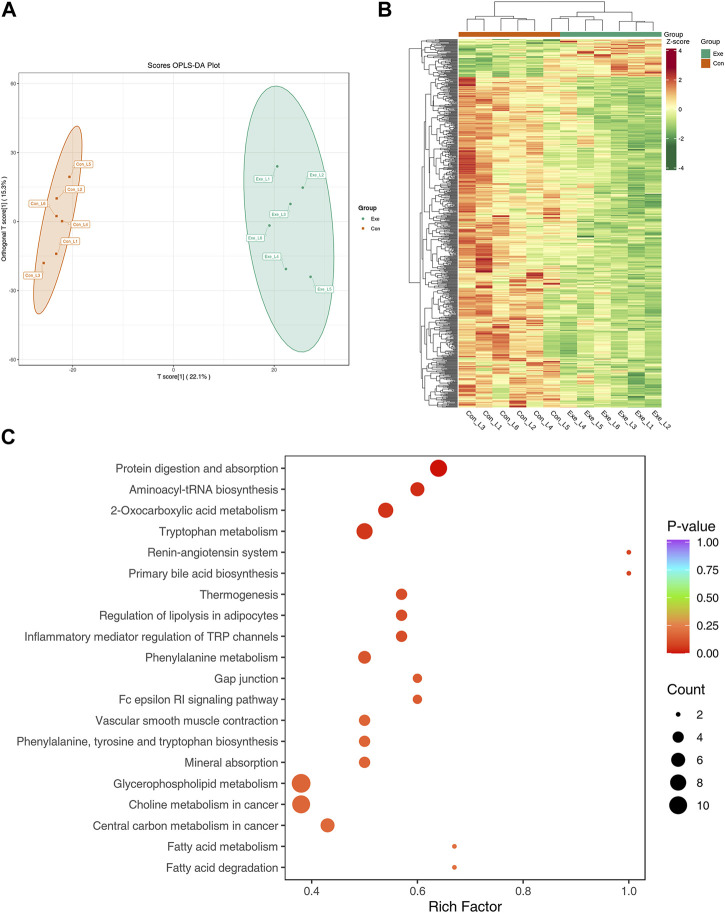
Metabolomics analysis in positive mode **(A)** OPLS-DA score plot of metabolome in positive mode. **(B)** Heatmap of the DEMs in positive mode. **(C)** KEGG pathway annotation for DEMs in positive mode.

In positive mode, the Orthogonal projections to latent structure-discriminant analysis (OPLS-DA) score plot exhibited distinct clusters between two groups (Figure 5A). Compared with Con group, 693 DEMs were identified, among which 71 metabolites were significantly increased, and 622 metabolites showed a downward trend in Exe group (Figure 5B). These metabolites included amino acids and their metabolites, glycerides, fatty acyl metabolites, carbohydrates and their metabolites, among which there were 8 free fatty acid metabolites, 11 glycerolipid metabolites and 43 glycerophospholipids, majority of which were low expressed in the Exe group. KEGG pathway analysis revealed that these 693 metabolites were enriched in inflammatory mediator regulation of TRP channels (Arachidonic acid, Capsaicin, 2-Dilinoleoyl-sn-glycerol), vascular smooth muscle contraction (20-Hydroxyeicosatetraenoic acid, arachidonic acid, 1,2-Dilinoleoyl-sn-glycerol, Norepinephrine), fatty acid degradation (trans-Oct-2-enoyl-CoA and Carnitine C16:0) and fatty acid metabolism (trans-Oct-2-enoyl-CoA and Carnitine C16:0) (Figure 5C).

**FIGURE 6:**
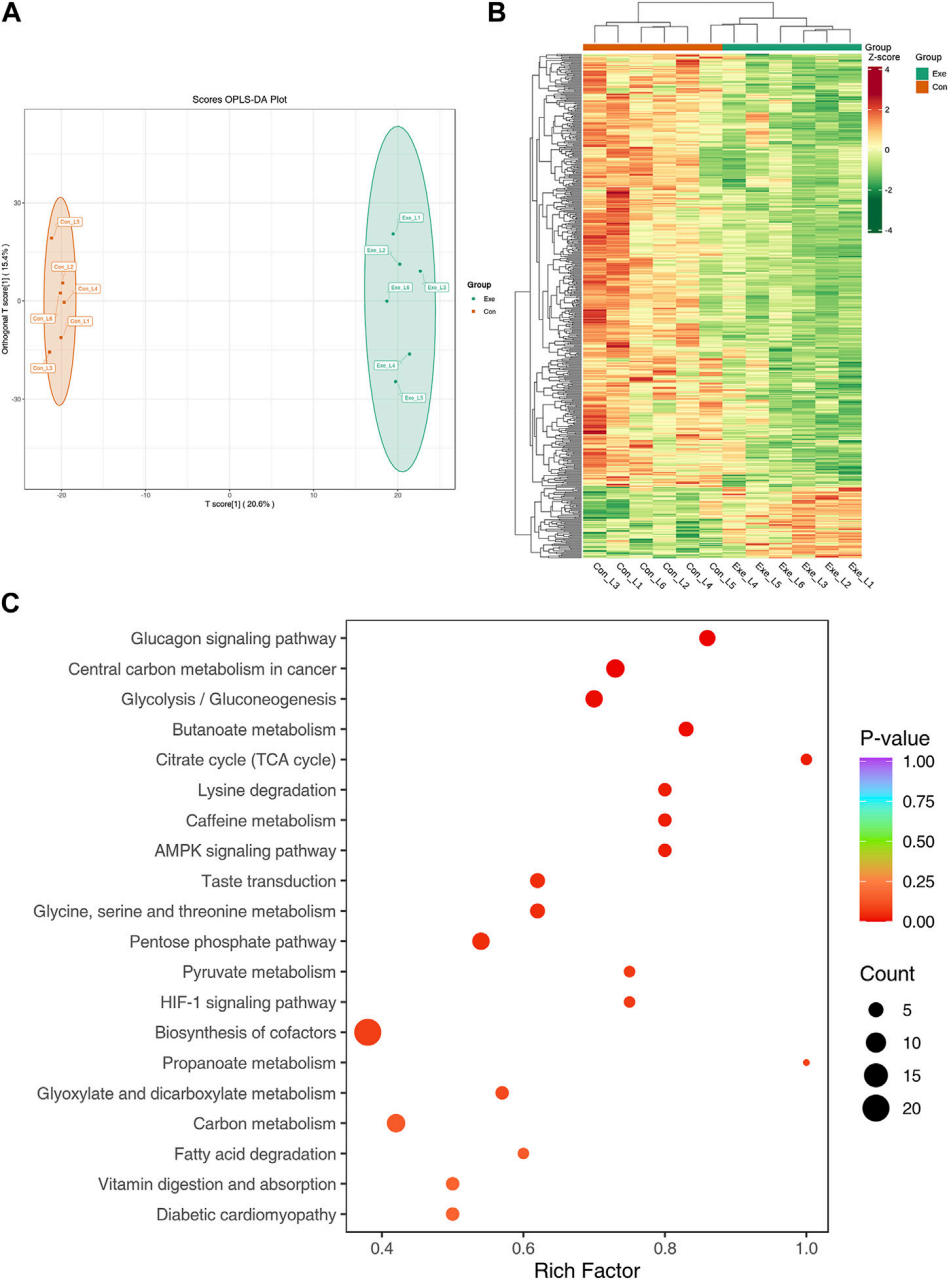
Metabolomics analysis in negative mode **(A)** OPLS-DA score plot of metabolome in negative mode. **(B)** Heatmap of the DEMs in negative mode. **(C)** KEGG pathway annotation for DEMs in negative mode.

In negative mode, the metabolomics analysis result showed that there were significant differences in the OPLS-DA score between the Con group and the Exe group (Figure 6A). Compared with Con group, 537 DEMs were screened in the Exe group, including 76 upregulated and 461 downregulated (Figure 6B). There were 3 free fatty acid metabolites, 8 glycerolipid metabolites and 15 glycerophospholipids, majority of which were low expressed in the Exe group. KEGG pathway analysis revealed that these 537 metabolites were enriched in citrate cycle (TCA cycle) (acetyl coenzyme A, phosphoenolpyruvate, cis-aconitic acid), HIF-1 signaling pathway (glucose, L-lactic acid, acetyl coenzyme A), fatty acid degradation (coenzyme A, acetyl coenzyme A, glutaric acid), AMPK signaling pathway, and glycolysis/gluconeogenesis (Figure 6C).

### 3.6 Correlation analysis between proteome and metabolome

**FIGURE 7:**
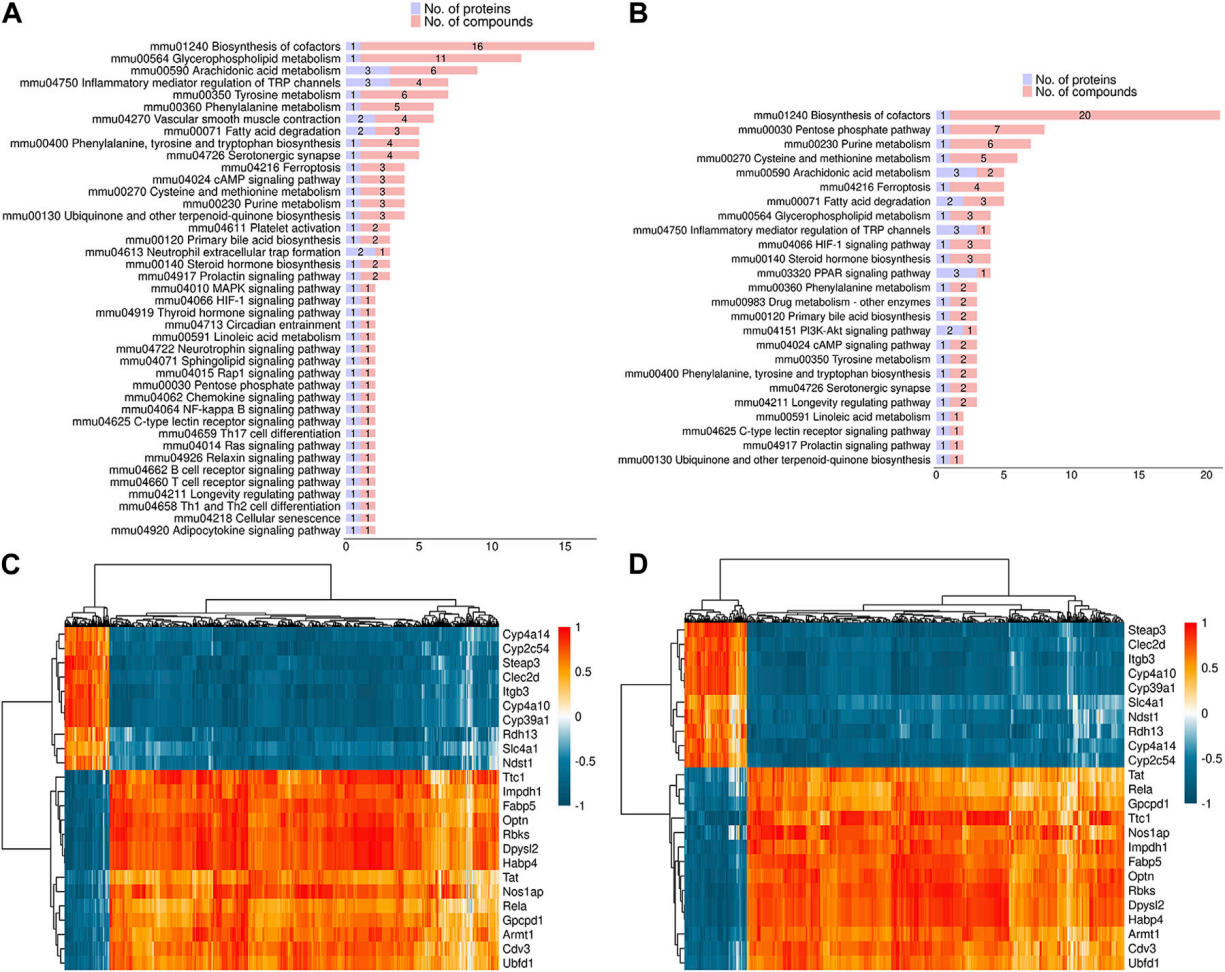
Correlation analysis between DEPs and DEMs **(A,B)** The common pathways in both DEPs and DEMs in positive **(A)** and negative mode **(B)**. **(C,D)** Spearman correlation analysis between DEPs and DEMs in positive **(C)** and negative mode **(D)**.

In order to investigate the potential association between proteome and metabolome, the overlapping pathways in both DEPs and DEMs were identified. As shown in Figure 7A, fatty acid degradation, vascular smooth muscle contraction, HIF-1 signaling pathway, inflammatory mediator regulation of TRP channels, and glycerophospholipid metabolism were common pathways in both DEPs and DEMs in positive mode. As shown in Figure 7B, fatty acid degradation, HIF-1 signaling pathway, inflammatory mediator regulation of TRP channels, glycerophospholipid metabolism, and PPAR signaling pathway were common pathways in both DEPs and DEMs in negative mode. In addition, spearman correlation coefficient was used to evaluate the correlation between DEPs and DEMs. The results indicated that 1,2-Dilinoleoyl-sn-glycerol was negatively associated with Cyp4a14 and Cyp4a10, 20-Hydroxyeicosatetraenoic acid was negatively associated with Cyp4a10, and arachidonic acid was negatively associated with Optn in positive mode (Figure 7C); acetyl coenzyme A was negatively associated with Cyp4a14 and Cyp4a10, acetyl coenzyme A and L-lactic acid were positively associated with Optn in negative mode (Figure 7D).

## 4 Discussion

Although exercise has potential benefits effects on systemic metabolism, its biological mechanisms is not sufficiently known. The current study is the first study to provide a comprehensive molecular characterization of the liver tissue in Exe and Con mice at the transcriptomic, proteomic, acetyl-proteomics, and metabolomic levels. In total, 88 DEGs, 25 DEPs, 185 DAPs, 207 DASs, and 693 DEMs in positive mode and 537 DEMs in negative mode were identified between Con and Exe group. In addition, the correlation analysis between transcriptome and proteome, and proteome and metabolome was also conducted to explore the possible regulatory relationships among the various omics data.

Peroxisome proliferation-activated receptors (PPARs) are fatty acid receptors that control the transcription of target genes, and all three of its isotypes (PPARα, PPARβ/δ and PPARγ) bind lipids and control lipid homeostasis in the liver (Montagner et al., 2016). The CYP4A subfamily are cytochrome P450 (P450) fatty acid hydroxylases that contribute to the ω-hydroxylation of fatty acids and participate in the metabolism of fatty acids (Nyagode et al., 2014). It is well known that Cyp4a10 and 4a14, the members of CYP4A subfamily, were the classic downstream target genes of PPARα. PPARα, expressed highly in hepatocytes, is a classic regulator for lipid and glucose metabolism (Xu et al., 2022). The activation of PPARα is beneficial to improve systemic fatty acid homeostasis and energy balance (Luo et al., 2016; Montagner et al., 2016). Specifically, the energy sensor PPARα is activated with lipids such as fatty acid, dimerizes with RXR to form PPAR-RXR complex and then upregulates the expression of PPARα target genes (i.e., Cyp4a14 and Cyp4a10), which serves a role in fatty acid degradation, oxidation, transport, synthesis, lipolysis, gluconeogenesis, autophagy and so on (Montagner et al., 2016; Wang et al., 2021; Xu et al., 2022). Notably, the upregulated Cyp4a14 and Cyp4a10 in the liver are responsible for catalyzing the ω-hydroxylation of arachidonic acid and medium-chain fatty acid, indicating an increased fatty acid oxidation efficiency, inhibition of lipid accumulation and increased demand for energy (von Hardenberg et al., 2018; Xu et al., 2011; Fu and Klaassen, 2014; Wu et al., 2020). Previous mice study indicated that fasting upregulated the mRNA relative expression content of PPARα, Cyp4a14 and Cyp4a10 in liver, which regulated glycerol use for gluconeogenesis to fulfill the increased demand for energy of the whole body and contributed to systemic fatty acid homeostasis (Montagner et al., 2016). In congruence with previous reports, the current study found high expressed hepatic Cyp4a10 and Cyp4a14 were the only two proteins that exhibited consistent expression trends at both the transcription level and the protein level and were involved in fatty acid degradation and arachidonic acid metabolism. PPAR signaling pathway was activated in liver of Exe mice as indicated by significant upregulation of Cyp4a14 and Cyp4a10. To the authors’ knowledge, this phenomenon is demonstrated for the first time in both the transcriptome and proteome, suggesting that chronic exercise induces complex changes in fatty acid turnover and the induction of PPAR alpha is consequent to the changes in fatty acid delivery to the liver, with Cyp4a10 and Cyp4a14 and other PPAR alpha target genes being induced downstream of PPAR alpha activation.

The eukaryotic proteome contains hundreds of different types of posttranslational modifications, among which acetylation is one of the major types with multiple effects on protein levels and metabolome levels (Drazic et al., 2016). Acetylation affects protein functions through a variety of mechanisms, including regulation of protein stability, enzyme activity, subcellular localization, and control of protein-protein and protein-DNA interactions (Narita et al., 2019). Protein acetylation usually occurs in two distinct forms, acetylation at their N^α^-termini of the nascent polypeptide chains and acetylation at the ε-amino group of lysine (Drazic et al., 2016). Lysine ε-acetylation is a major feature in the regulation of mitochondrial metabolism and 63% of proteins localized to mitochondria contain lysine acetylation sites (Baeza et al., 2016). Acetyl-proteomics analysis detected 185 DAPs, of which 44 proteins were mitochondrial proteins, such as Acsl1, Acadvl, Mdh2, and Aco2, involved in lipid, amino acid and vitamin metabolism. Acsl1, as a PPARα target or co-activator, converts free long-chain fatty acids into fatty acyl-CoA esters, and thereby plays a key role in lipid biosynthesis and fatty acid degradation (Thering et al., 2009). Lu et al. (2014) indicated that high expression of Acsl1 implied activation of PPARα, which enhanced fatty acid metabolism through increasing fatty acid binding and co-activation. Acadvl catalyzes the first step of the mitochondrial fatty acid beta-oxidation pathway. It has been well-recognized that Mdh2 catalyzes the reversible oxidation of malic acid to oxaloacetic acid in the TCA cycle. Aco2 catalyzes the interconversion of citrate to isocitrate via cis-aconitate in the second step of the TCA cycle. Taken together, acetyl-proteomics analysis also uncovered the effect of chronic exercise on liver tissue metabolism in mice.

It has been well known that physical activity has beneficial effects on lipid metabolism (Shanmugam et al., 2020). The metabolomics analysis indicated a notably different metabolome between Exe group and Con group in both positive mode and negative mode. These metabolites included amino acids and their metabolites, glycerides, fatty acyl metabolites, carbohydrates and their metabolites, and were involved in metabolic pathways such as fatty acid metabolism, citrate cycle and glycolysis/gluconeogenesis. Correlation analysis between proteome and metabolome uncovered multiple important interactions between metabolites and proteins. For instance, arachidonic acid was negatively associated with Optn, acetyl coenzyme A was negatively associated with Cyp4a14 and Cyp4a10, and acetyl coenzyme A was positively associated with Optn. Acetyl-coenzyme A is an important chemical in the human body and a source of carbon for the synthesis of lipoic acid, cholesterol and ketone bodies. It is generated in various metabolic processes, including lipid metabolism, ketone body metabolism, amino acid catabolism, and fatty acids metabolism (Trefely et al., 2020). By influencing the acetylation profile of several proteins, Acetyl-coenzyme A controls key cellular processes, including energy metabolism, mitosis, and autophagy, both directly and via the epigenetic regulation of gene expression (Pietrocola et al., 2015). Arachidonic acid is an essential fatty acid and an important factor for the function of all cells, particularly in the nervous system, immune system, and vascular endothelium (Kursun et al., 2022). It has a variety of physiological functions, which is a component of the phospholipid bilayer of cell membranes, a regulator of gene expression, a vasodilator/vasoconstrictor, and also plays an important role in the nervous system (Zhou et al., 2021).

Based on the results of transcriptomic, proteomics, acetyl-proteomics and metabolomics analysis, chronic moderate intensity exercise has certain effects on liver metabolism and protein synthesis in mice. Chronic moderate intensity exercise may participate in liver energy metabolism by influencing the expression of Cyp4a14, Cyp4a10, arachidonic acid and acetyl coenzyme A and regulating fatty acid degradation, arachidonic acid metabolism, fatty acyl metabolism and subsequent acetylation.

## Data Availability

The datasets presented in this study can be found in online repositories. The names of the repository/repositories and accession number(s) can be found below: The transcriptomics data have been deposited to the GSE229755 dataset (GSE229755, https://www.ncbi.nlm.nih.gov/geo/query/acc.cgi?acc=GSE229755). The mass spectrometry proteomics data have been deposited to the ProteomeXchange Consortium (http://proteomecentral.proteomexchange.org) via the iProX partner repository with the dataset identifier PXD041538 (https://proteomecentral.proteomexchange.org/cgi/GetDataset?ID=PXD041538). Metabolomics data have been deposited to the EMBL-EBI MetaboLights database (DOI: 10.1093/nar/gkz1019, PMID: 31691833) with the identifier MTBLS7609 (https://www.ebi.ac.uk/metabolights/MTBLS7609).
